# Biomarkers in Inflammatory Bowel Disease-Associated Spondyloarthritis: State of the Art and Unmet Needs

**DOI:** 10.1155/2019/8630871

**Published:** 2019-05-30

**Authors:** Devis Benfaremo, Michele Maria Luchetti, Armando Gabrielli

**Affiliations:** Clinica Medica, Dipartimento di Scienze Cliniche e Molecolari, Università Politecnica delle Marche, Ancona, Italy

## Abstract

Inflammatory bowel disease-associated spondyloarthritis is a systemic disease characterized by the chronic inflammation of both the gastrointestinal tract and the musculoskeletal system. Since inflammatory bowel disease-associated spondyloarthritis has been associated with a significant diagnostic delay, which may lead to poor quality of life and progression of joint damage, efforts to discover new reliable and noninvasive diagnostic biomarkers have been made. We reviewed the state of the art of biomarker research in inflammatory bowel disease-associated spondyloarthritis, showing that to date it has been largely unsatisfactory. Only a few of the biomarkers that have been investigated are likely to enter the clinical practice upon further validation in independent cohorts. The research of new and innovative biomarkers for inflammatory bowel disease-associated spondyloarthritis is warranted.

## 1. Introduction

Inflammatory bowel disease-associated spondyloarthritis (SpA/IBD) is a systemic disease characterized by the chronic inflammation of both the gastrointestinal tract and the musculoskeletal system [1]. From the rheumatologist's point of view, SpA/IBD is included in the group of spondyloarthritides (SpA), together with ankylosing spondylitis (AS), reactive arthritis, undifferentiated arthritis, and psoriatic arthritis [2]. In fact, inflammatory bowel diseases (IBD), namely, Crohn's disease (CD) and ulcerative colitis (UC), are among the most frequent extra-articular complications that may occur in patients with AS. From the gastroenterologist's perspective, arthritis is the most frequent extraintestinal manifestation in IBD and may develop before, simultaneously with, or after the diagnosis of overt intestinal disease [3].

The prevalence of IBD in patients with AS is estimated between 5 and 10%, but nearly 50% of AS patients have subclinical gut inflammation [4]. From the point of view of IBD, 3% of the patients have concomitant AS and 13% have peripheral SpA according to a recent meta-analysis [5], but radiographic sacroiliitis, either symptomatic or subclinical, may involve half of the IBD patients [6].

The fact that joint symptoms may be mild or absent and the use of concomitant immunosuppressive therapies for IBD and the use of the New York criteria for AS may hamper the early diagnosis of SpA/IBD, resulting in a significant diagnostic delay, has been associated with several adverse outcomes for the patient, including poor quality of life and progression of joint damage [7, 8].

Evidence from preclinical studies corroborated the hypothesis that IBD and SpA may share a common pathogenesis, as in both diseases there is an involvement of tumor necrosis factor (TNF-*α*) and interleukin (IL) 23/17 pathways [9]. If the involvement of TNF-*α* is well known and further attested by the long experience of treatment with TNF inhibitors for both SpA and IBD, clinical trials of anti-IL17A agents in IBD failed to reach the primary endpoint and even appear to have a worsening effect on CD [10]. Conversely, ustekinumab, the first IL-12/23 inhibitor, is now approved for the treatment of CD but failed to improve symptoms and signs of axial SpA [11].

Taken together, these differences suggest that, despite the several features that SpA and IBD have in common, the coexistence of joint and gut inflammations is unique. This is further suggested by the proportion of human leukocyte antigen- (HLA-) B27-positive patients in the axial SpA/IBD group, far lower than AS and SpA in general [3, 12, 13]. Moreover, asymptomatic sacroiliitis, which is present in a significant percentage of IBD patients, is not associated with HLA-B27 [12]. Finally, the coexistence of gut and joint involvements advocates the multidisciplinary management of SpA/IBD patients, like in another multifaceted SpA like psoriatic arthritis [14].

Overall, SpA/IBD may be not only a subset of the broad entities of IBD and SpA but also a distinct and rather peculiar disease requesting a tailored clinical evaluation and therapeutic approach. For such an accomplishment, referral strategies such as the use of screening questionnaires [15] and the identification of simple biomarkers are warranted.

## 2. What Are Biomarkers?

A biomarker is a “characteristic that can be objectively measured and evaluated as an indicator of a normal biologic process, a pathophysiologic process, or a pharmacologic response to a therapeutic intervention” [16].

Ideal biomarkers should be sensitive, specific, reproducible, and derived from a noninvasive procedure. Each biomarker could theoretically be useful for the processes of diagnosis, treatment response, and prognosis evaluation, but such instruments are rare in clinical practice.

A further differentiation should be made between molecular, imaging, and clinical biomarkers of disease. Molecular biomarkers are biochemical variables that can be measured in the blood, stools, and other fluids or tissues of the human body. Objective, quantitative measurements of molecular biomarkers through a variety of techniques serve as indicators of normal or pathologic processes or indicators of response to therapy. Of note, the availability of new sequencing technologies allowed the identification of newer genetic biomarkers of disease [17].

Imaging technologies, such as MRI, CT scans, and ultrasound, can be regarded as biomarkers when they are used for the evaluation of disease activity and response to treatment. Imaging methods allow structural and functional assessments of disease activity and therapy.

Clinical biomarkers are physical signs and symptoms that may contribute to the diagnosis and assessment of established disease, but they are rarely followed by a game-changing decision making.

Biomarkers can further be divided into descriptive and mechanistic. Descriptive biomarkers reflect the state of a disease but are not directly involved in disease pathogenesis, whereas mechanistic biomarkers participate in the biologic mechanisms of disease. If descriptive biomarkers provide limited diagnostic and prognostic information, mechanistic biomarkers, reflecting the dysregulation of molecular pathways directly involved in pathogenesis of the disease, are more useful for guiding clinical decision making [18].

Several biomarkers have already been studied in SpA and IBD, but specific biomarkers addressing the coexistence of gut and joint inflammations, respectively, in SpA and IBD patients are lacking.

In this review, we will summarize the state of the art of biomarker research in SpA/IBD, trying to highlight lights and shadows of every tool that has been endorsed. Unless stated otherwise, we will primarily consider biomarkers that may be helpful to diagnose or identify SpA/IBD among patients with IBD or SpA.

## 3. Overview of Biomarkers in SpA/IBD

### 3.1. Genetic Biomarkers

A genetic biomarker is a DNA sequence that causes disease or is associated with susceptibility to disease.

To date, a variety of genetic loci that increase susceptibility to AS have been identified.

HLA-B27 is the prototype of genetic biomarkers in SpA, but several other nonmajor histocompatibility complex (MHC) loci like endoplasmic reticulum aminopeptidase 1 (ERAP-1), IL-23R, lymphotoxin beta receptor (LTBR), and TNFRSF1A (tumor necrosis factor receptor 1) have been described [19, 20].

HLA-B27 is present in about 85–95% of patients with AS in the US, Europe, and China. However, within a population, only 5% of HLA-B27-positive individuals develop AS or another form of SpA [20].

In addition to being a risk factor for SpA, HLA-B27 is likely implicated in the pathogenesis of AS by several mechanisms which include arthritogenic peptide theory, noncanonical HLA dimerization, HLA-B27 misfolded response, or alteration of gut microbiome by HLA-B27 [21].

As already pointed out, the role of HLA-B27 positivity in predicting SpA development in IBD patients is questionable, given the lower prevalence in SpA/IBD populations [3, 12, 13]. More recently, in a Norwegian cohort of IBD patients followed up to 20 years, the prevalence of HLA-B27 among IBD patients with AS was 57.1%, confirming the lower prevalence than in AS without IBD [22]. However, the presence of HLA-B27 was associated with an increased occurrence of inflammatory back pain, axial SpA, and AS. In this study, the quite high frequency of HLA-B27 in the Norwegian population probably contributed to the higher prevalence of AS and axial SpA in IBD patients (4.5% and 7.7%, respectively) [22].

Coming to IBD, the first CD susceptibility gene that has been identified is CARD15, also known as NOD2. Variants within this gene increase the risk for CD by threefold for heterozygous individuals and 33-44-fold for homozygous and compound heterozygous individuals [23]. Disappointingly, several studies excluded an association between CARD15 variants and AS or SpA in IBD populations [22, 24–26]. However, in SpA patients, an association was found between the carriage of CARD15 variants and the development of chronic subclinical gut inflammation, with an OR of 2.9 as compared to control population and of 5.8 as compared to SpA patients without gut inflammation [27].

In a Turkish study, the ERAP1 (rs26653) polymorphism was found to increase the disease risk in patients with AS and IBD compared with the control group (OR 2.6 for both groups). The results of the study also suggest that ERAP1 (rs26653) polymorphism may be an important genetic factor influencing the pathogenesis of UC with axial SpA (OR 2.9).

By contrast, IL-23R gene polymorphisms seem to have a protecting role in both IBD (OR 0.38 and 0.73 for CD and UC, respectively) and AS (OR = 0.53 − 1.27) [28–30].

Several other common risk variants for CD and AS have been described, but their significance should be evaluated in independent studies [31].

### 3.2. Biochemical Biomarkers

Biochemical markers are soluble molecules that may serve as an aid in diagnosing or in predicting susceptibility to the disease, monitoring disease activity, and predicting response to treatment and relapse. They can be measured in blood, urine, stools, or other body fluids or tissues.

Traditional serum markers of inflammation such as C-reactive protein (CRP) and erythrocyte sedimentation rate (ESR) are not useful for the diagnosis of SpA in IBD patients and vice versa, since they lack both sensitivity and specificity [21]. This is not surprising, since inflammation may originate from both gut and joints. Moreover, the proportion of SpA and IBD patients that display abnormal CRP levels is variable, and it is not unusual to find patients with active disease and normal values of ESR and CRP. As a result, the serum concentrations of CRP are not significantly different between SpA/IBD and IBD patients [32].

Of note, in the proportion of SpA patients in which they are elevated (30-50%), CRP serum levels may be useful to assess disease activity [33] and to predict response to treatment [34] and radiographic progression [35]. CRP has therefore been included in the Ankylosing Spondylitis Disease Activity Score (ASDAS-CRP), which is currently used to assess disease activity in SpA. Also, in IBD, CRP levels have been correlated with higher disease activity and response to treatment [36, 37]. Therefore, even if the measurement of CRP is not useful for the diagnostic evaluation of SpA/IBD, it could be used to monitor disease activity and response to treatment.

Several cytokines have been measured in the serum of patients with either SpA or IBD, and their potential use as biomarkers has been evaluated.

Since IL-6 is the main driver of CRP production, it is not surprising that higher serum levels of IL-6 have been described in both SpA and IBD patients, though the clinical utility of serum IL-6 measurement is uncertain. In fact, IL-6 serum levels could not discriminate between SpA/IBD and IBD patients, as did not several other cytokines, like IL-10, IL-21, IL-22, IL-23, and interferon gamma (IFN-*γ*) [32]. Nevertheless, in SpA/IBD patients, a moderate positive correlation was found between serum concentrations of IL-23 and clinical disease activity of SpA [32]. In another small study, the authors evaluated the serum levels of IL-23 in 26 IBD vs. 11 SpA/IBD patients and found that IL-23 was significantly higher in SpA/IBD (67.73 ± 40.85 pg/ml) compared to IBD patients (37.15 ± 10.37 pg/ml) [38]. Another study, found a weak association between elevated IL-1 alpha and its receptor antagonist and SpA/IBD [39]. To date, there is no convincing evidence that inflammatory markers and cytokines could be used as disease biomarkers in SpA/IBD.

Several serological antibodies have been studied in IBD, including anti-*Saccharomyces cerevisiae* (ASCA), perinuclear anti-neutrophil cytoplasmic antibodies (pANCA), anti-I2, anti-*Escherichia coli* outer membrane porin (anti-OmpC), and anti-flagellin (anti-CBir1), but only ASCA and pANCA showed meaningful accuracy for the diagnosis of CD and UC, respectively [40].

In an early study conducted in patients with SpA, ASCA IgA levels, but not ASCA IgG, were higher than in control groups, but they were not related to the presence of gut inflammation. Conversely, ASCA IgG were found to be strongly associated with CD. The authors conclude that ASCA IgG may serve as a biomarker of CD in AS patients, though they did not include SpA/IBD patients in their evaluations [41].

Two later studies confirmed that ASCA IgA are higher in SpA patients than healthy controls [42, 43], whereas a third study failed to observe such an association [44]. In a recent study, ASCA levels seem to be associated with higher disease activity in a cohort of SpA patients [45].

Mundwiler et al. studied serum from 80 AS patients and 80 controls assessing for ASCA, anti-I2, anti-OmpC, anti-CBir1, and ANCA. They found no difference in positivity rates between AS and control groups with the established IBD values. Significantly more AS patients had ASCA IgG (26% vs. 13%), ASCA IgG and IgA (27% vs. 12%), and anti-I2 (25% vs. 14%) [46]. Another study failed to replicate these findings and reported higher levels of ANCA, but not ASCA, anti-I2, anti-OmpC, and anti-CBir1, in AS patients [47].

To assess the utility of these IBD-related biomarkers in SpA/IBD patients, De Vries et al. enrolled 179 patients (52 with AS, 50 with UC, 51 with CD, and 26 with IBD and AS). pANCA, ASCA (IgA and/or IgG), and OmpC antibodies were found in 21%, 30%, and 19% of the AS patients, respectively, but only pANCA could be considered a predictor of UC in AS patients (OR 8.2) [48].

Conversely, Wallis et al. could not replicate these results and found that among 76 patients with AS, 77 patients with AS/IBD, and 48 patients with mechanical back pain, SpA/IBD patients demonstrated a higher prevalence of ASCA, anti-OmpC, and anti-CBir1, but not ANCA, when compared to AS alone [49]. Overall, these studies provided conflicting results, and to date, these antibodies have no role in the assessment of SpA/IBD.

Fecal calprotectin is one of the most extensively studied stool markers in IBD. In a meta-analysis, the accuracy of fecal calprotectin to differentiate IBD and non-IBD patients was exceptionally high (AUC 0.95-0.98 using a cut-off level of 50 *μ*g/g and 100 *μ*g/g, respectively) [50].

Moreover, in IBD patients, fecal calprotectin decreased upon treatment and may predict disease relapse [51–53].

Several studies reported elevated levels of fecal calprotectin in patients with SpA. Abnormal fecal calprotectin is found in around 40-70% of the SpA patients and seems to correlate with articular disease activity [47, 54, 55]. However, only a minority of the SpA patients with elevated fecal calprotectin actually exhibited either clinical or subclinical gut disease [55].

In addition, the measurement of serum calprotectin, another marker of inflammation, provided conflicting results. Whereas some studies found significantly higher serum calprotectin in AS patients compared to healthy controls [56–58], other did not [55]. Even if serum calprotectin could be a predictor of radiographic progression [59], its specificity and sensitivity are too poor to be transferred in the clinical practice [58].

With regard to the possible use of calprotectin as a biomarker in SpA/IBD, Klingberg et al. designed a longitudinal study studying 164 AS patients after a 5-year follow-up and found that baseline fecal calprotectin was directly correlated with SpA disease activity at the 5-year follow-up. Moreover, fecal calprotectin could predict the development of CD (cumulative incidence 1.5% at 5 years) [55]. The latter study is encouraging, but further longitudinal studies are certainly needed to prove the role of serum and fecal calprotectin as gut disease biomarkers in SpA patients.

The identification of biomarkers of articular involvement in IBD patients is more intriguing and difficult at the same time. SpA and AS are traditionally considered seronegative diseases, and in recent years, considerable efforts have been made to discover reliable biomarkers of disease, with little or no success [60]. Even if a variety of biomarkers have been investigated, only a few showed potential diagnostic accuracy in order to be transferred in the clinical practice after being validated in independent cohorts.

Promising candidate biomarkers of SpA, other than CRP and cytokines, include the following: vascular endothelial growth factor (VEGF), matrix metalloproteinase-3 (MMP-3), Dickkopf-1 (DKK-1), sclerostin (SOST), and anti-CD74 antibodies.

VEGF levels are elevated in patients with AS and axial SpA and seem to correlate with radiographic progression [61, 62].

Higher MMP3 levels have been shown to reflect disease activity and treatment response in SpA, though the results among the studies are inconsistent [63, 64].

The Wnt family consists of a number of small secreted glycoproteins involved in regulation of a variety of cellular activities with critical roles during development [65]. The Dickkopf family, which includes DKK-1, inhibits the Wnt pathway. DKK-1 serum levels have been described as being lower in most [66–68] but not all studies [69] conducted in AS patients. However, DKK-1 levels appear to be inversely correlated with radiographic progression.

Sclerostin (SOST) is another inhibitor of the Wnt pathway that has been extensively studied in SpA.

The majority of the studies report lower serum levels of SOST in AS patients compared to controls [70–74], with a significant inverse correlation between SOST levels and radiographic progression, but other studies failed to confirm these findings [69, 75]. Furthermore, Tsui et al. previously reported the detection of higher levels of anti-SOST IgG in patients with AS [76].

Recently, Baerlecken et al. reported the detection of high serum levels of CD74 IgG in SpA patients [77]. The authors analyzed 145 sera from 94 axial SpA and 51 non-SpA patients, reporting that anti-CD74 antibodies were detected in 85.1% in axial SpA but in only 7.8% in non-SpA patients and their sensitivity and specificity for diagnosing axial SpA were, respectively, 85.1% and 92.2% [78]. Unfortunately, the diagnostic value of anti-CD74 antibodies has been recently questioned by an independent study that observed a low specificity of anti-CD74 when used for diagnostic purposes in early axial SpA, even if they confirmed the presence of higher serum levels in SpA patients compared to controls [79].

Only a few of these biomarkers, borrowed from AS, have been also evaluated in SpA/IBD.

YKL-40 (also known as Chitinase 3-like 1) is a glycoprotein produced by inflammatory, cancer, and stem cells. An old report identified YKL-40 as a possible biomarker for SpA/IBD [80]. In this study, serum YKL-40 was measured in 171 patients, 29 PsA, 66 IBD, and 76 SpA/IBD. The authors observed significant differences in YKL-40 values in SpA/IBD patients compared to IBD patients without joint involvement. In particular, YKL-40 was higher in SpA/IBD than IBD patients and healthy controls. The AUC for YKL-40 was 0.82, superior to that of CRP.

In another study, serum antibodies against anti-mutated citrullinated vimentin (anti-MCV) and second-generation anti-cyclic citrullinated peptide (anti-CCP2) antibodies were measured in 125 IBD patients, 35% of which had SpA/IBD [81]. Disappointingly, the proportion of anti-MCV and anti-CCP2 positivity was similar between IBD patients with or without articular involvement.

A more recent study reported that serum SOST and anti-SOST IgG levels may be useful to detect axial SpA in IBD patients [74]. Luchetti et al. measured serum SOST and anti-SOST levels in 85 SpA/IBD patients, 40 IBD patients, and healthy controls. Patients affected by SpA/IBD with axial involvement displayed significantly lower levels of SOST and higher levels of anti-SOST-IgG compared to patients with only peripheral arthritis, IBD, and controls. Moreover, SOST and anti-SOST-IgG serum levels were inversely correlated and associated with the duration of articular symptoms. Both biomarkers showed good accuracy in predicting the presence of axial SpA in patients with IBD (AUC 0.88 and 0.84 for SOST and anti-SOST, respectively).

In recent years, significant alterations in the intestinal microbiome of both IBD and SpA patients have been extensively reported [82, 83]. Early studies in AS observed an increased frequency of anti-*Klebsiella pneumoniae* antibodies in the serum of both SpA and IBD patients [84–87], but the clinical significance of these findings is uncertain.

Recently, the SpA/IBD microbiome has been studied using a novel technique, which couples the sorting of IgA-coated microbiota with 16S ribosomal RNA (rRNA) sequencing (called IgA-seq), focusing the analysis only on microbiota identified by the immune system [88]. Viladomiu et al. observed a selective enrichment in IgA-coated *Escherichia coli* in patients with SpA/CD compared to CD alone. These bacteria were similar to adherent-invasive E. coli (AIEC) pathotype. The authors could also demonstrate that colonization of germ-free mice with SpA/CD-derived E. coli isolates induced T helper 17 (Th17) cell mucosal immunity, providing evidence of a mechanistic link between intestinal microbiota and systemic inflammation.

Table 1 summarizes the properties of the candidate biochemical markers that have been evaluated in SpA/IBD to date. Overall, none of them possess the characteristics of the perfect biomarker, i.e., accuracy, reproducibility, and noninvasivity, though SOST and anti-SOST serum levels are promising tools for the assessment of axial disease in IBD patients. Other antibodies (such as ASCA and pANCA) and fecal calprotectin may be useful to suspect IBD in SpA patients, but all of them need further validation in well-designed studies.

### 3.3. Clinical Biomarkers

Clinical associations may contribute to the suspicion of extraintestinal and/or extra-articular manifestations, but they are not reliable as disease biomarkers.

Documented clinical associations between AS (or SpA) and IBD include the link between higher intestinal disease activity and the development of peripheral arthritis [89, 90], though a recent study with a longer follow-up failed to confirm this finding [91]. Conversely, patients reporting persistent or relapsing intestinal disease activity over a 20-year IBD course seemed to be more prone to developing axial SpA [22], even though this finding was quite unexpected, since axial SpA was thought to progress independently of the intestinal disease activity [92, 93].

Articular disease has been further independently associated more with CD than UC [5], with female gender [94, 95], with older age [89], and with smoking [90].

In patients with AS, development of IBD has been significantly associated with markers of increased articular disease activity but to some extent also with worse physical function and worse patient global well-being at the time of diagnosis of IBD [96].

In another larger study conducted in 1250 axial SpA patients, the development of IBD was associated with disease duration, with an increase of the risk by 30% per 10 years of disease duration [97].

In the Esperanza cohort, IBD was associated with peripheral SpA more than axial SpA [98].

### 3.4. Imaging Biomarkers

Imaging biomarkers are image features that should be obtained by noninvasive techniques and should be relevant for the diagnosis, the assessment of disease activity, or the prediction of outcomes.

Ultrasound is a noninvasive imaging technique that is increasingly being used to assess SpA patients, especially for the diagnosis of enthesitis, which is common in IBD patients.

An Italian study group found that ultrasound abnormalities of the entheses are present in a high proportion of IBD patients without clinical signs and symptoms of SpA. Of the 81 patients, 71 (92.6%) presented almost one tendon alteration, including higher thickness, enthesophytosis, bursitis, and erosions. However, power Doppler was positive only in 13/81 (16%) patients. Furthermore, ultrasound enthesopathy was not associated with activity, duration, and type of gut disease [99].

The early diagnosis of axial involvement is essential in SpA/IBD, since the diagnostic delay is associated with poor outcomes. The prevalence of radiographic sacroiliitis is elevated in IBD [6, 100], but radiographic alterations are known to occur late in the natural history of SpA. Magnetic resonance imaging (MRI) is now the reference standard for the assessment of nonradiographic sacroiliitis. MRI colonography or enterography is also increasingly used to assess disease activity and complications in IBD. This imaging technique may have a role in the assessment of sacroiliitis in the same MRI session. A retrospective study performed on 186 IBD patients found that the prevalence of inflammatory sacroiliitis on MRI, performed for the evaluation of the intestinal disease, was 16.7%. Sacroiliitis was bilateral in 14 cases and unilateral in 17 cases. Older age and female gender were significantly associated with the presence of sacroiliitis. Other factors such as type of IBD, disease duration and localization of IBD, history of surgery, CRP, intestinal disease activity, and treatment were not associated with sacroiliitis [101].

Since systematic colonoscopy assessment demonstrated a mucosal inflammation characteristic of CD in up to one-third of patients with SpA, Kopylov et al. examined the hypothesis if video capsule endoscopy (VCE) could be superior to detect inflammatory bowel lesions in patients with SpA.

In the SpACE Capsule Study, 64 adult SpA patients underwent VCE and standard colonoscopy with biopsies. Small bowel inflammation was present in 42.2% vs. 10.9% of patients according to VCE and standard colonoscopy, respectively. Interestingly, no correlation was observed with the presence of intestinal symptoms and CRP [102].

## 4. Conclusions

The prominent features of SpA/IBD, such as the lower prevalence of HLA-B27, the higher proportion of female patients, and the differential response to treatment of joint and gut diseases, suggest that this is a rather peculiar entity, distinct from AS, which deserves to be properly investigated, particularly with the goal to reduce unnecessary diagnostic delay and achieve an earlier diagnosis. This target may be accomplished using accurate biomarkers of disease, but, to date, the quest for biomarkers in SpA/IBD has been quite neglected and largely unsatisfactory. In this review, we showed that only a few of the biomarkers that have been investigated are likely to enter the clinical practice upon further validation in independent cohorts. The research of new and innovative biomarkers of SpA/IBD is therefore warranted.

## Figures and Tables

**Table 1:** Properties of candidate biochemical markers for inflammatory bowel disease-associated spondyloarthritis.

Reference	Candidate biomarker	Sampling	Population	Findings	Notes
Hoffman et al. [41]	ASCA (IgA and/or IgG)	Serum	26 patients with CD, 108 patients with SpA, 56 patients with RA, and 45 healthy controls	ASCA IgA levels similar between patients with SpA with and without histologically proved gut inflammation	ASCA IgA levels were raised in AS and uSpA vs. RA and HC (AUC 0.72)

Bernardi et al. [80]	YKL-40	Serum	171 patients: 29 PsA, 66 IBD (36 CD and 30 UC), and 76 SpA/IBD (44 CD/32 UC)	YKL-40 levels significantly higher in SpA/IBD vs. IBD patients (AUC 0.82)	

De Vries et al. [48]	pANCA	Serum	179 patients: 52 with AS, 50 with UC, 51 with CD, and 26 with IBD+AS	pANCA associated with UC in AS patients (OR 8.2, 95% CI 1.2–55.6)	pANCA detected in 21% of AS patients
	ASCA (IgA and/or IgG)	Serum	179 patients: 52 with AS, 50 with UC, 51 with CD, and 26 with IBD+AS	ASCA found in 40% of SpA/CD vs. 30% of AS and 90% of CD patients	Not useful
	OmpC antibodies	Serum	179 patients: 52 with AS, 50 with UC, 51 with CD, and 26 with IBD+AS	OmpC Ab found in 30% of SpA/IBD patients and 19% of AS patients	Not useful

Al-Jarallah et al. [81]	Anti-MCV	Serum	125 IBD patients (44 of which with SpA/IBD) vs. 81 healthy controls	Anti-MCV positivity similar between IBD and HC (16.8% vs. 16.0%) No discrimination between SpA/IBD and IBD	Not useful
	Anti-CCP2	Serum	125 IBD patients (44 of which with SpA/IBD) vs. 81 healthy controls	Anti-CCP2 positivity similar between IBD and HC (6.4% vs. 6.2%) No discrimination between SpA/IBD and IBD	Not useful

Wallis et al. [49]	ASCA IgG and IgA	Serum	76 patients with AS, 77 patients with AS/IBD, and 48 patients with mechanical back pain	ASCA IgG higher in AS/IBD than AS patients (14% vs. 0%)	
	Anti-OmpC	Serum	76 patients with AS, 77 patients with AS/IBD, and 48 patients with mechanical back pain	Anti-OmpC higher in AS/IBD than AS patients (27% vs. 12%)	
	Anti-CBir1	Serum	76 patients with AS, 77 patients with AS/IBD, and 48 patients with mechanical back pain	Anti-CBir1 higher in AS/IBD than AS patients (42% vs. 20%)	
	ANCA	Serum	76 patients with AS, 77 patients with AS/IBD, and 48 patients with mechanical back pain	ANCA not significantly different between AS/IBD and AS patients	Not useful

Klingberg et al. [55]	Calprotectin	Feces	164 AS patients followed up for 5 years	The development of CD (3 pts) predicted by baseline high fecal calprotectin > 266 mg/kg (AUC 0.91) No incident cases of UC in the AS population	Fecal calprotectin was elevated (>50 mg/kg) in 70% pts at the baseline Globally, 7.4% of patients had signs of inflammation in the large or small intestine

Viladomiu et al. [88]	IgA-coated E. coli	Fecal microbiome	59 IBD patients with or without peripheral SpA	IgA-coated E. coli enriched in CD-SpA vs. CD alone	CD-SpA E. coli isolates induced Th17 mucosal immunity

Luchetti et al. [74]	SOST	Serum	85 pts with axial or peripheral SpA/IBD Control groups (IBD, AS, RA, and HC)	Lower levels in AxSpA/IBD vs. PerSpA/IBD and control groups (AUC 0.88)	Lower levels of SOST correlate with higher levels of anti-SOST-Ig
	Anti-SOST antibodies	Serum	85 pts with axial or peripheral SpA/IBD Control groups (IBD, AS, RA, and HC)	Higher levels in AxSpA/IBD vs. PerSpA/IBD and control groups (AUC 0.84)	Higher levels of anti-SOST-Ig correlate with lower levels of SOST
